# Chromophore-Assisted Light Inactivation for Protein Degradation and Its Application in Biomedicine

**DOI:** 10.3390/bioengineering11070651

**Published:** 2024-06-26

**Authors:** Lvjia Zhou, Jintong Na, Xiyu Liu, Pan Wu

**Affiliations:** 1 State Key Laboratory of Targeting Oncology, National Center for International Research of Bio-Targeting Theranostics, Guangxi Key Laboratory of Bio-Targeting Theranostics, Collaborative Innovation Center for Targeting Tumor Diagnosis and Therapy, Guangxi Medical University, Nanning 530021, China; zhoulvjia@sr.gxmu.edu.cn (L.Z.); najintong@sr.gxmu.edu.cn (J.N.); 2 School of Pharmacy, Guangxi Medical University, Nanning 530021, China

**Keywords:** CALI, protein degradation, biomedicine, photosensitizer

## Abstract

The functional investigation of proteins holds immense significance in unraveling physiological and pathological mechanisms of organisms as well as advancing the development of novel pharmaceuticals in biomedicine. However, the study of cellular protein function using conventional genetic manipulation methods may yield unpredictable outcomes and erroneous conclusions. Therefore, precise modulation of protein activity within cells holds immense significance in the realm of biomedical research. Chromophore-assisted light inactivation (CALI) is a technique that labels photosensitizers onto target proteins and induces the production of reactive oxygen species through light control to achieve precise inactivation of target proteins. Based on the type and characteristics of photosensitizers, different excitation light sources and labeling methods are selected. For instance, KillerRed forms a fusion protein with the target protein through genetic engineering for labeling and inactivates the target protein via light activation. CALI is presently predominantly employed in diverse biomedical domains encompassing investigations into protein functionality and interaction, intercellular signal transduction research, as well as cancer exploration and therapy. With the continuous advancement of CALI technology, it is anticipated to emerge as a formidable instrument in the realm of life sciences, yielding more captivating outcomes for fundamental life sciences and precise disease diagnosis and treatment.

## 1. Introduction

Proteins are the executor of most life activities, and their expression influences many biological functions, such as catalysing biochemical reactions, transporting molecules across membranes, promoting cell growth and division, and facilitating cell adhesion and migration. Investigating protein activity levels and their interactions is vital for comprehending the molecular mechanisms underlying cell differentiation, signaling pathways, disease progression, and clinical diagnosis [1]. As proteomics research continues to expand its scope and depth, tumor proteomics has gained significant prominence in cancer studies [2]. Particularly within precision oncology, proteomics holds great potential for accurately characterizing tumors clinically, facilitating more precise cancer diagnoses, and enhancing treatment strategies for cancer patients [3].

The primary challenge in cellular proteomics research has been how to carry out accurate localization and function studies in living cells. Presently, it is customary to induce abnormal expression of cellular proteins via gene knockout or RNA interference techniques, when aiming to explore protein function. However, transcriptional regulation alone does not comprehensively influence protein expression due to the intricate involvement of post-translational modifications, subcellular localization dynamics, and protein–protein interaction networks. Furthermore, manipulating the expression of protein levels through gene-level regulation introduces a time delay and may elicit other compensatory mechanisms that are difficult to detect and regulate effectively, thereby potentially compromising the accuracy of protein function investigations. In addition, it has been reported that small molecule compounds such as PROteolysis TArgeting Chimeras (PROTACs) can effectively utilize the proteasome to degrade target proteins through ubiquitination by an E3 ligase complex [4]. Meanwhile, by incorporating azobenzene photoswitches into PROTACs, Reynders et al. have designed photoswitchable versions, which they named PHOtochemically TArgeting Chimera [5]. These small molecules have little or no proteolytic ability in the dark but can be activated with blue-violet light (380 to 440 nm). They can be used to degrade a variety of targets like BRD2–4 and FKBP12 so as to achieve the purpose of artificial precise light control to target inactivated proteins. However, existing methods utilizing small molecule compounds to induce and control protein inactivation suffer from limitations such as time-consumption, high costs, inadequate subcellular localization, insufficient target specificity, potential off-target effects, and cytotoxicity. These drawbacks have hindered their widespread application in live-cell proteomics research. Therefore, we need a simple, fast, target-specific technology that can be precisely controlled to inactivate the target protein.

**Figure 1:**
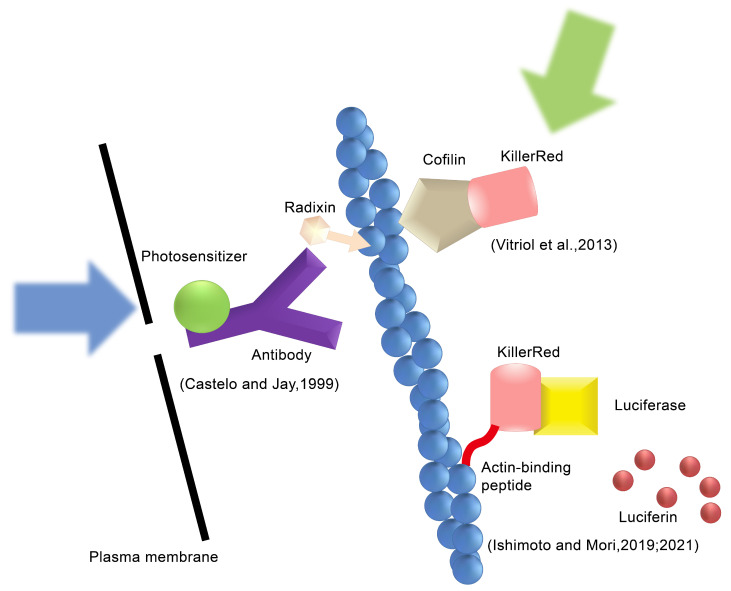
Types of the CALI method. The type 1 CALI method, Castelo and Jay use an anti-radixin antibody labeled with a photosensitizer and generate ROS by applying excitation light [8]. The type 2 CALI method, Vitriol et al. fuse KillerRed with cofilin and express it in cells to induce ROS action on F-actin [9]. Ishimoto et al. generate ROS using the luciferin–luciferase reaction instead of excitation by external light [10,11]. Reproduced with permission from [12]. Copyright© 2022 Ishimoto and Mori.

Chromophore-assisted light inactivation (CALI) is a technology which mainly induces targeting protein inactivation through light control [6]. Distinguished by the chromophore, CALI can be categorized into two main approaches at present (Figure 1). The first approach is to introduce a photosensitizer to the target protein through an antibody or chemically synthesized protein tag; the second approach is to use genetic engineering technology to modify the gene for the protein of interest to co-express a fluorescent protein as a fusion protein with the target protein. Furthermore, due to the continuous evolution of chromophores, CALI has progressed from initially being laser-activated to being light-activated; as such, based on the structure and properties of chromophores, LED light sources (blue and green), ultraviolet light sources, and even ordinary white light can be selected as excitation light sources. After labeling the target protein with a photosensitizer, light is applied to induce the generation of reactive oxygen species (ROS), which subsequently attack and inactivate the target protein. From initially selectively inactivating alkaline phosphatase or β galactosidase by binding an antibody conjugated with malachite green [6], investigating the distinct roles of Fasciclin I and II in grasshopper pioneer neuron development [7], CALI has been extensively used in many medical biological experiments, which serves as compelling evidence for its promising potential as a cutting-edge technology that has garnered significant attention within the field of biology in recent years. This review provides an overview of the principles, key points (chromophores and labeling techniques), and applications of CALI technology based on an analysis of pertinent articles from PubMed. It also discusses the latest developments of the key points, with the aim of increasing readers’ understanding of CALI technology and promoting its development and application.

## 2. Mechanism of CALI Targeting Inactivated Proteins

**Figure 2:**
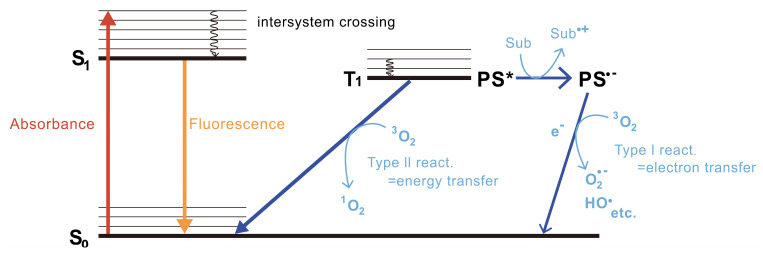
The principles of reactive oxygen species (ROS) generation. In the Type I reaction, the generated photosensitizer radical anion (PS^•−^) then transfers an electron to oxygen to produce ROS such as superoxide radicals (O_2_^•−^) and hydroxyl radicals (HO^•^). In contrast, the Type II reaction directly produces singlet oxygen (^1^O_2_) by energy transfer from the T1 PS* to oxygen. Reproduced with permission from [22]. Copyright© 2021 The Japan Academy.

CALI was initially hypothesized to mediate photoinduced thermal denaturation by the photosensitizer’s return from the excited state to the ground state after laser irradiation excitation and to inactivate its proximate proteins. However, later thermal diffusion calculations indicated that the thermal energy generated by the photosensitizer was too low to cause thermal denaturation of proximate proteins. Moreover, employing a ROS quencher can effectively inhibit the inactivation of proteins by photosensitizers. Consequently, it can be inferred that protein inactivation in CALI is primarily induced by oxygen radicals produced by the photosensitizer following laser irradiation [13]. Currently, it is widely acknowledged that there exist two primary mechanisms responsible for the generation of ROS induced by photosensitizers (Figure 2) [14]. First, when the photosensitizer is irradiated with a specific wavelength of light, it is activated and jumps up from the ground state energy state (S0) to the unstable second excited energy state (S2), then it quickly undergoes internal conversion (IC) to jump down to a lower unstable first excited energy state (S1), and finally it quickly jumps from the S1 to a more stable and long-lasting excited triplet state (T1), through the intersystem crossing process (ISC) [15,16]. If a photosensitizer in the T1 state reacts with nearby biological substrates through electron transfer to generate free radicals, these free radicals further interact rapidly with biomolecules, resulting in ROS such as superoxide radicals and hydroxyl radicals, which are type I reactions. Alternatively, if the photosensitizer in the T1 state does not produce electron transfer, the photon energy is instead transferred directly to triplet ground state molecular oxygen (^3^O_2_); ^3^O_2_ quickly jumps to a singlet excited state and becomes singlet state oxygen ^1^O_2_, which are type II reactions. The occurrence of electron–hydron transfer and type I and/or type II reactions depends on the redox potential between the photosensitizer and the nearby substrate [17]. Meanwhile, if the distance between the photosensitizer and the functional domain of the target protein exceeds the diffusion distance of ROS, the objective of damaging or inactivating the protein will not be achieved. To ensure high spatiotemporal accuracy of CALI, it is generally recommended that the effective distance of ROS be within 1–5 nm [18,19,20], which can effectively achieve inactivation of the target protein without exceeding the average distance of protein–protein interactions within cells (8 nm) [21]. Despite its many attractive features, CALI has not been widely utilized due to inherent limit.

## 3. Photosensitizers of CALI

Photosensitizers are substances that can be irradiated with a specific wavelength of light, absorbing and transmitting photon energy to produce ROS within living organisms. These photosensitizers find applications not only in CALI but also in various biooptical fields such as photopolymerization, photodegradation, photocatalysis, and photodynamic therapy [23]. Over the years, extensive research and exploration have led to the successful development of numerous photosensitizers by humans. Currently, these photosensitizers can be broadly categorized into chemically synthesized ones and genetically encoded variants. Based on this classification system for photosensitizers, CALI can also be preliminarily classified into gene-expression-based approaches and non-gene-expression-based strategies.

### 3.1. Chemical Photosensitizers (CPSs)

The initial application of CALI technology was CPS malachite green [6]. In this report, Jay employed malachite green-labeled streptavidin to bind with a biotinylated enzyme, inactivating alkaline phosphatase and β-galactosidase, respectively. Conversely, unbiotinylated alkaline phosphatase and β galactosidase did not exhibit significant inactivation under these conditions. In the meantime, he also used a malachite green-labeled dye-conjugated antibody to acetylcholinesterase for the purpose of inactivating acetylcholinesterase located on the extracellular surface of human erythrocytes [24]. Notably, within the same solution, significant inactivation of acetylcholinesterase was observed, while alkaline phosphatase remained unaffected, and there were no observable changes in the morphology of the human erythrocytes. These findings suggest that malachite green attached to a specific ligand or antibody can selectively target and deactivate specific proteins without causing damage to other proteins or cells. Although this study provided an innovative optical manipulation technique for biological research and demonstrated the feasibility of CALI technology, malachite green was not quickly popularized at that time due to malachite green’s requirement of a high energy pulsed laser power, which was not suitable for general microscopy.

Subsequent investigations have revealed that using fluorescein instead of malachite green can permit users to CALI-like processes on general microscopes and microscope bulbs [25]. Surrey et al. pioneered the application of CALI technology on a simple in vitro system consisting of microtubules and motor proteins, comparing the malachite green of standard CALI with fluorescein-labeled hemagglutinin antibody fragments and low-power continuous-wave light illumination. They examined the effectiveness of CALI targeted to kinesin, demonstrating that the use of fluorescein with ordinary continuous light sources can destroy kinesin activity without affecting structural formation elsewhere [26]. Subsequently, Beck et al. found that a fluorescein-labeled β-galactosidase scFv monoclonal antibody inactivated bacterial β-galactosidase using different light sources [27]. They found that whether laser light, diffuse monochromatic light, or even a 60-watt light bulb was used fluorescein effectively inactivated bacterial β-galactosidase, a similar effect as is seen in CALI biological studies using bacterial galactosidase [13,26]. These results are enough to prove that fluorescein is more applicable than malachite green in CALI technology, but the fluorescein efficiency in producing ROS is insufficient, and there remains room for further improvement in this aspect.

**Figure 3:**
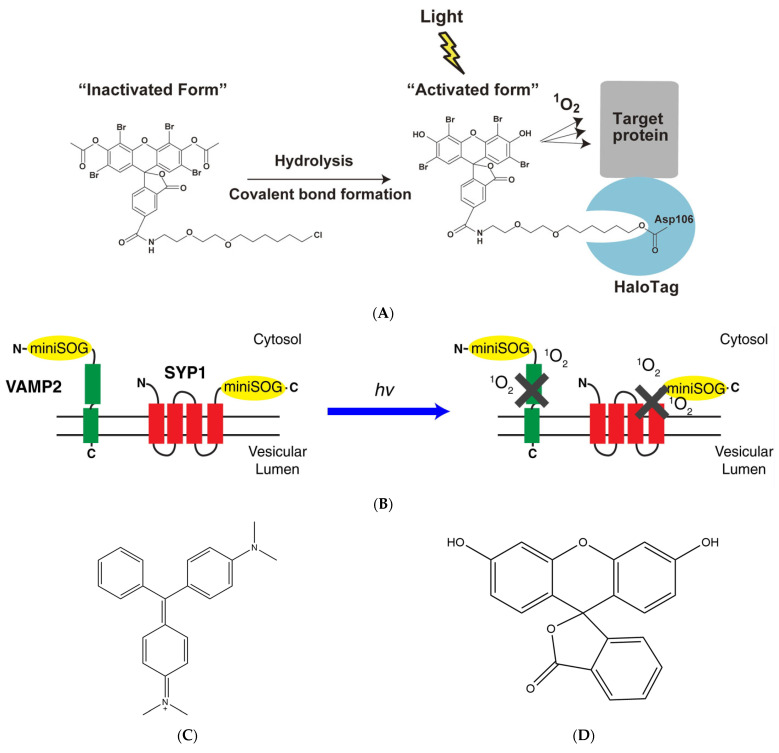

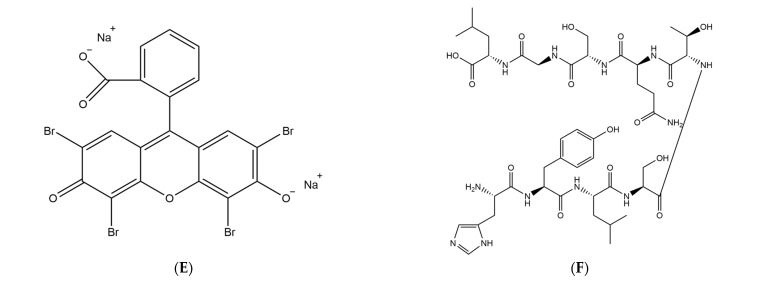
Different categories of photosensitizers employ distinct methodologies to label target proteins. (**A**) Targeting eosin to the protein of interest with the HaloTag labeling technology, reproduced with permission from [22], Copyright© 2021 The Japan Academy. (**B**) The two designs of CALI-based synaptic inactivation system with miniSOG fused to Vesicular Associated Membrane Protein 2 (VAMP2) or synaptophysin (SYP1), reproduced with permission from [44], Copyright© 2013 Elsevier Inc. (**C**–**F**) Chemical structure of malachite green, fluorescein, eosin, and GFP.

Later, numerous novel photosensitizers were synthesized based on fluorescein. Eosin, for example, is a brominated xanthene derivative of fluorescein in an alcoholic medium, exhibiting an 11-fold higher singlet oxygen generation capacity compared to fluorescein. Additionally, upon illumination, eosin generates a substantial amount of detectable fluorescence enabling the identification of protein binding through fluorescence microscopy techniques. Then, it was confirmed that eosin-labeled β-galactosidase antibodies exhibited a 5-fold higher efficiency in the inactivation of β-galactosidase compared to fluorescein-labeled antibodies [18,22], Takemoto et al. successfully synthesized a membrane-permeable eosin ligand with eosin and HaloTag, resulting in the development of an eosin labeling system with high cell permeability and target specificity (Figure 3A). HaloTag7, a second-generation mutant of halogenated alkane dehalogenase, is fused at the N-termini of Protein Kinase C-gamma (PKC-γ) and labeled with eosin. PKC-γ labeled by HaloTag7 and eosin was translocated to the cytoplasmic membrane within 5–15 min after TPA (12-O-Tetradecanoylphorbol 13-acetate) stimulation. However, upon irradiation with a strong green light at 520–560 nm, the translocation of PKC-γ could not be detected on the cytoplasmic membrane, indicating that eosin-labeled HaloTag-based CALI is feasible for inactivating PKC-γ. Subsequently, Takemoto et al. used eosin-labeled CALI to inactivate the Aurora B kinase resulting in complete cessation of cell division or formation of multinuclear structures post-cell division, while unlabeled cells remained unaffected. These findings collectively demonstrate that eosin exhibits a high rate of ROS production and can be utilized for CALI using conventional microscopy techniques.

### 3.2. Genetically Encoded Photosensitizers (GEPSs)

The characteristics of CPSs are their high efficiency in generating ROS and, when connecting with antibody or protein tagging technology, better application of CALI to various proteins within living cells. However, the use of CPSs can introduce additional chemicals into living cells, potentially triggering other cellular compensation mechanisms or cytotoxicity. Moreover, complete removal of CPSs from cells poses a challenge. To address these issues, an alternative approach could involve producing the photosensitizer through translation of the cell’s own genetic code. GEPSs, designed using genetic engineering methods, can artificially regulate their expression in specific cell populations and subcellular locations and times by adding regulatory sequences. GEPSs are proteins that can be completely removed from cells by proteolysis [28], and due to the reduction in chemical treatment of cells, the integrity of cell activity and function can be better maintained compared with CPSs [29].

The first fluorescent protein discovered, green fluorescent protein (GFP), is a 238-amino acid protein with high fluorescence quantum yield, which exhibits green fluorescence upon excitation by blue light to ultraviolet light, but has weak photosensitizing activity; hence, applying it to CALI technology is ineffective [19]. Researchers subsequently developed a gene-encoded red fluorescent protein called KillerRed, which is severalfold more active than GFP and has successfully become the first GEPS for CALI. KillerRed is a fluorescent protein derived from the non-fluorescent hydrozoan jellyfish anm2CP [30]; screening genetic mutation and introduction of amino acids were used to improve its phototoxicity and ROS production efficiency. KillerRed predominantly exhibits type I reactivity, generating superoxide radicals and a limited amount of singlet oxygen when irradiated with green light, thereby demonstrating potent cytotoxicity. However, when employing KillerRed for protein inactivation purposes, KillerRed is a dimeric molecule [31], which may lead to detrimental effects on mitochondria, cell membranes, and DNA and interfere with cell mitosis, eventually causing cell cycle arrest or cell death [32,33]. Henceforth, due to these concerns regarding its potential adverse consequences on cellular components and processes, KillerRed has not gained widespread utilization as a photosensitizer for CALI applications.

To address the issue of KillerRed, Takemoto et al. successfully obtained a monomeric protein called SuperNova by replacing six amino acids in KillerRed [34]. SuperNova exhibits nearly identical structural features and photosensitizing activity to KillerRed, while demonstrating enhanced folding and maturation efficiency in vitro, manifesting as a monomeric protein under physiological conditions. Comparative analysis with KillerRed revealed that SuperNova does not impede normal cell mitosis. Subsequently, Takemoto et al. constructed a cofilin–SuperNova fusion protein based on actin turnover in living mammalian cells. This fusion protein was transiently expressed in living COS7 cells, and upon intense orange light irradiation, it effectively suppressed the motility of actin filaments qualitatively. In contrast, actin filaments remained unaffected when not labeled by the cofilin–SuperNova fusion protein [35,36]. These findings are consistent with previous studies on the regulation of cofilin-mediated actin dynamics and demonstrate the superiority of SuperNova over KillerRed for CALI applications.

KillerOrange is another orange dimer variant obtained through random mutagenesis in KillerRed [37]. Unlike KillerRed, KillerOrange’s fluorescence is derived from tryptophan and can be excited by blue-green light. However, despite maintaining all the photophysical characteristics similar to KillerRed, KillerOrange exhibits a propensity for dimer formation and demonstrates cytotoxicity upon blue-green light excitation. Consequently, its suitability for CALI technology is limited. Riani et al. [38] introduced a V44A-containing mutation around the KillerOrange chromophore to synthesize a green monomer variant named SuperNova-Green, which is the first green photosensitizer protein derived from KillerRed. Subsequently, they used SuperNova-Green and SuperNova to inactivate the pleckstrin homology domain (PHdomain) of phospholipase C-delta 1 (PLC-δ1). Upon excitation with 560 nm light, it was observed that the SuperNova-labeled PH domain underwent significant inactivation; however, no substantial inactivation was observed for SuperNova-Green. Conversely, when excited with 440 nm light, an opposite outcome was obtained. These findings suggest that both SuperNova-Green and SuperNova can be effectively applied to CALI alone or in combination for selective intracellular inactivation of diverse proteins. Recently, Gorbachev et al. [39] introduced mutant S10R into SuperNova and KillerRed through random mutagenesis and directed optimization, synthesizing the second generation of SuperNova and KillerRed (named SuperNova2 and KillerRed2), which produce enhanced brightness, chromophore maturity, and phototoxicity in bacterial and mammalian cell cultures. These advancements enable researchers to conduct phototoxicity experiments using lower light doses and shorter exposure times while serving as valuable templates for further mutagenesis to generate novel genetically encoded photosensitizers.

The LOV (light-oxygen-voltage) sensing domain flavin mononucleotide (FMN) of phototactic proteins [40,41] is a photosensitizer capable of producing singlet oxygen [42,43]. By conducting saturating mutagenesis on the associated cysteine (Cys426) of the LOV2 domain of Arabidopsis phototropin 2 (AtPhot2) and its nearby binding site, Shu et al. [29] successfully generated a novel protein called miniSOG (mini Singlet Oxygen Generator). Comprising only 106 amino acid residues, miniSOG is the smallest monomeric protein that emits green light. Notably, it exhibits a higher rate of singlet oxygen production compared to the aforementioned photosensitive proteins. Furthermore, miniSOG functions as a monomeric fluorescent protein without inducing abnormal cell division. Lin et al. (Figure 3B) [44] successfully achieved precise inhibition of hippocampal neurotransmitter release by genetically fusing miniSOG with the SNARE protein complex VAMP2, which is located at the presynaptic terminal of neurons, and synaptophysin (SYP1). Additionally, when expressing miniSOG-labeled VAMP2 in Caenorhabditis elegans and subjecting them to light irradiation, a decrease in movement was observed with some individuals becoming completely paralyzed. However, approximately 2–3 h after removing the light stimulus, recovery of movement could be observed in *C. elegans*. These findings underscore the potential utility of miniSOG as a valuable tool for precise modulation of synaptic activity. By utilizing light-induced inactivation of the VAMP2 complex, both in vivo and in vitro, they have demonstrated the applicability of miniSOG for light-mediated synaptic inhibition. The categories and characteristics of photosensitizers are summarized in Table 1. The chemical structure of malachite green, fluorescein, eosin, and GFP are summarized in Figure 3C–F.

## 4. CALI Photosensitizer Labeling Targeted Protein Technology

Since the pioneering application of malachite green by Jay in 1988, CALI technology has developed tremendously. These progressions can be attributed not only to the continuous enhancement of CALI’s dyes in terms of their photosensitivity and activity but also to the development of targeted labeling techniques for specific proteins using photosensitizers. Different categories of photosensitizers employ distinct methodologies to label target proteins. Chemical photosensitizers utilize techniques such as antigen and antibody labeling or protein tag labeling, while gene-coding photosensitizers rely on genetic engineering approaches.

### 4.1. CPS Protein Labeling

Jay combined malachite green with isothiocyanate groups for the first time in vitro [6]. The isothiocyanate group can react with the amino terminal or primary amino group of the protein in neutral buffer to target malachite green to the protein. However, this is only effective for inactivating proteins in vitro and does not achieve the purpose of targeting proteins for inactivation intracellularly. Subsequently, Diefenbach et al. [45] inactivated myosin II(MII) by combining malachite green with isothiocyanic acid, addressing the role of myosin MII in chick dorsal root ganglion neuronal growth cone motility. Higurashi et al. [46] combined malachite green with isothiocyanic acid, inactivating collapse response mediator protein CRMP1 or CRMP2. Either CRMP1 or CRMP2 inactivation in the neurite shaft led to arrested neurite outgrowth, and their study successfully elucidated the distinct roles played by CRMP1 and CRMP2 in arresting axonal cell growth. Similarly, isothiocyanate group labeling is also suitable for fluorescein [47]. In addition to isothiocyanate groups, researchers have labeled monoclonal antibodies with photosensitizers in order to achieve targeted binding of specific proteins. For instance, Hoffman-Kim et al. [48] employed malachite green-labeled monoclonal antibody MAb 327 to effectively inhibit the tyrosine kinase pp60c-src, resulting in a remarkable twofold or higher increase in overall neurite extension rate, proving that pp60c-src is a negative regulator of lamin-1-mediated neurite outgrowth in chicken dorsal root ganglion sensory neurons. Additionally, employing a modular protein labeling system based on synthetic ligands, covalent binding of photosensitizers to target proteins can be achieved in solution or living cells. The covalent bonds formed are usually highly specific and respond rapidly under physiological conditions. For example, Griffin et al. [49] designed a tetracysteine domain containing four closely spaced cysteines that can specifically bind to two trivalent arsenic compounds. Subsequently, they successfully synthesized a 4′,5′-bis(1,3,2-dithioarsolan-2-yl) fluorescein, which they called FLASH-EDT2 (fluorescein arsenical helix binder, bis-EDT adduct). This fluorescein is membrane-permeant, and it can enter the cell to specifically bind to the protein of interest containing the tetracystine domain by extracellular administration. Martin et al. [50] improved the affinity of fluorescein by optimizing the tetracysteine sequence, enabling binding to a much broader spectrum of cellular proteins within living cells. However, this labeling method has certain non-specific labeling and cytotoxicity problems and can only be used to label recombinant proteins that are expressed at very high levels [51]. Subsequently, Keppler et al. [52,53] proposed a method based on the human DNA repair protein O^6^-alkylguanine-DNA alkyltransferase (hAGT) fusion protein. hAGT can irreversibly transfer the alkyl group on the substrate O^6^-alkylguanine-DNA to one of its cysteine residues. The hAGT’s substrate specificity is relatively low, and it can also achieve similar reactions with the nucleobase O^6^-benzylguanine (BG). So, they synthesized the BG derivatives BGBT, a diacetate of fluorescein. By combining hAGT with the target protein to form a fusion protein, BGAF can be applied in different organisms and used to incorporate a variety of labels. In addition, the protein tag HaloTag, designed by Los et al. [54] can be used for covalent binding with synthetic HaloTag ligands. Takemoto et al. [18] have successfully achieved CALI using the HaloTag system.

### 4.2. GEPS Protein Labeling

Unlike CPSs, GEPSs enable expression of fusion proteins by combining target proteins with the fluorescent protein through genetic engineering techniques. Fluorescent proteins are expressed through genetic coding, thereby effectively avoiding introduction of foreign reagents into cells. For example, Vitriol et al. [55] proposed an improved lentiviral system capable of simultaneously achieving the knockout and rescue of endogenous cellular proteins using EPFG fusion proteins. With this lentiviral system, they achieved CALI of EGFP-capping protein (CP) in rescued deficient cells and also achieved CALI of EGFP-Mena in rescued fibroblasts derived from Mena/VASP/mice. Qi et al. [56] genetically engineered *C. elegans* neurons to express a fusion protein of miniSOG and TOMM-20, which is the major receptor for the import of polypeptides into mitochondria [57]. Upon blue light irradiation, miniSOG caused neurons to die quickly without detectable damage to surrounding tissues.

## 5. Recent Applications of CALI in the Field of Biomedicine

With the progressive advancements of photosensitizers and protein labeling technologies, CALI technology has reached a state of maturity. From initial employment for target protein inactivation in solution and at cellular levels, CALI is now being increasingly utilized across various studies. This section presents an overview of the recent applications of CALI in the biomedical field, with the aim of enhancing our comprehension of the characteristics and advantages associated with this technology.

**Figure 4:**
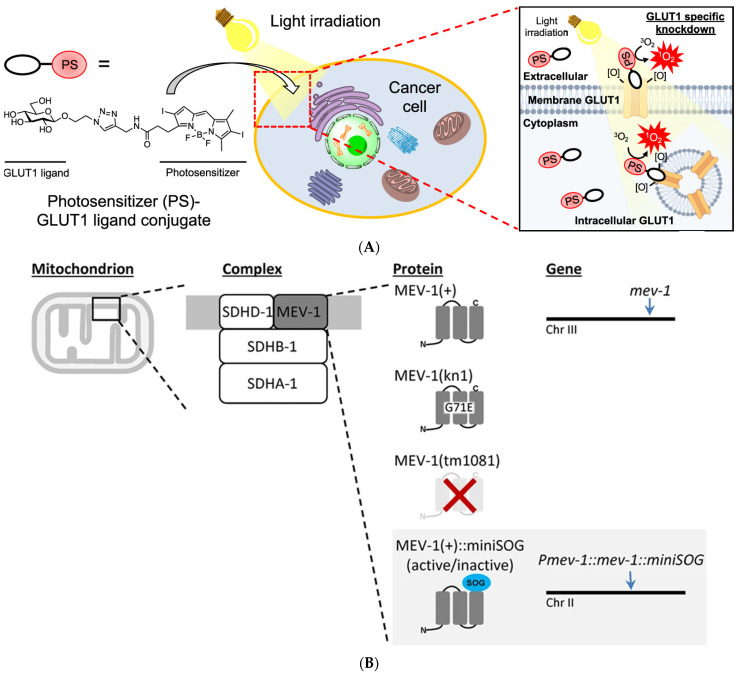

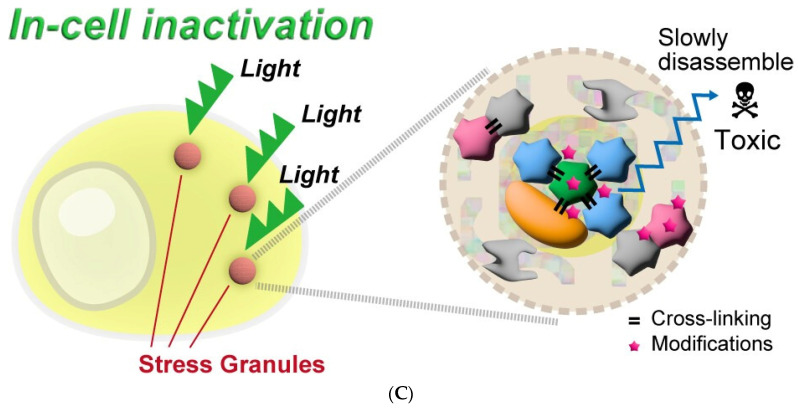
Recent application examples of CALI. (**A**) Conjugating photosensitizers with GLUT1 ligands to achieve inactivation of GLUT1, reproduced with permission from [65], Copyright© 2022, Kazuki Miura, Yijin Wen, Michihiko Tsushima and Hiroyuki Nakamura. (**B**) Genetically encoded photosensitizer miniSOG is used for CALI of mitochondrial respiratory chain complex II, reproduced under the terms of the CC-BY Creative Commons Attribution 4.0 International license (http://creativecommons.org/licenses/by/4.0/, accessed on 25 April 2024) from [58], Copyright 2016, Andrew P. Wojtovich, Alicia Y. Wei, Teresa A. Sherman, Thomas H. Foster and Keith Nehrke. (**C**) The inactivation of SGs by using a genetically encoded red fluorescence protein (SuperNova-Red) as a photosensitizer. Reproduced under the terms of the CC-BY Creative Commons Attribution 4.0 International license (http://creativecommons.org/licenses/by/4.0/, accessed on 25 April 2024) from [66], Copyright 2024 Koizumi, T.; Fujimoto, A.; Kawaguchi, H.; Kurosaki, T.; Kitamura.

*C. elegans*, a transparent multicellular eukaryotic organism, serves as an excellent animal model for bio-optics research, and as such, it was originally also used as an animal model for CALI. Mitochondria play a pivotal role in cellular functions by providing energy to cells. However, modifying the oxidative respiration-related genes of *C. elegans* to induce loss of function is mostly unfeasible. Wojtovich et al. (Figure 4B) [58] employed a Mos1-mediated single-copy insertion technique to introduce miniSOG as a fusion protein with the SDHC subunit of mev-1-encoded respiratory complex II in *C. elegans*, while blue-light irradiation was utilized to deactivate complex II within mitochondria. Manipulating ROS production through CALI technology will enhance our comprehension of mitochondrial function and oxidative respiratory chain activity at an organism-wide level, and miniSOG-mediated CALI has also become a novel genetic platform for acute inactivation of respiratory chain components. Subsequently, Trewin et al. [59] used CRISPR/Cas9 to fuse SuperNova with the C-terminus of mitochondrial complex II succinate dehydrogenase subunits B and C in *C. elegans* and demonstrated distinct physiological responses that generate equal amounts of ROS on both sides of the mitochondrial membrane within the mitochondrial complex-II microdomain by measuring the activity of key proteins such as NADPH and cytochrome C oxidase.

After obtaining satisfactory experimental results in *C. elegans*, CALI was successfully extended to Drosophila, a widely utilized genetic and evolutionary research model. The genital rotation of Drosophila involves the contraction and expansion of cell–cell junctions, with the formation of novel connections being essential for continuing and completing cell dynamics and tissue shape shaping; however, the underlying mechanisms of their growth remain unclear [60]. Uechi et al. [61] discovered that Drosophila myosin II plays a crucial role in the growth of junctions. Subsequently, they employed the green fluorescent protein SuperNova to label the endogenous regulatory light chain of myosin II in drosophila. Myosin II activity in labeled drosophila was normal, while it was inactivated after laser irradiation. Through CALI, they demonstrated that the adhesive transmembrane protein Sidekick promotes tricellular contacts movement by mediating myosin II-driven contraction and altering adhesion properties at tricellular contacts, leading to cell–cell junction extension during ongoing junction remodeling. Despite being a relatively novel technique in Drosophila research, CALI proves to be an invaluable tool for developmental genetics as it enables direct visualization of localized protein inactivation and its subsequent consequences on cellular dynamics.

Furthermore, Takemoto et al. [62] have successfully developed a monoclonal antibody targeting the extracellular domain of GluA1 and labeled it with the photosensitizer eosin. After injecting this labeled antibody into the dorsal hippocampal CA1 region of mice, the fear memory acquired by mice is eliminated by inactivating synaptic GluA1 homomeric CP-AMPA receptors by light. Rama et al. [63] devised a photosensitive V0c subunit to enable real-time monitoring of synaptic transmission between interconnected CA3 pyramidal cells in organotypic section slices and subsequently validated the role of V-ATPase V0c subunit (ATP6V0c) in neurotransmitter release downstream of synaptic vesicle acidification through CALI. Wavreil et al. [64] successfully inactivated target proteins of interest within the plant cortex using CALI with a miniSOG and selectively delayed or inhibited asymmetric cell division. Miura et al. (Figure 4A) [65] achieved photodynamic therapy development by using GLUT1 as a target protein of CALI to assess the protein inactivation capacity of several photosensitizers. Koizumi et al. (Figure 4C) [66] formed a fusion protein by labeling the G3BP1 and TDP-43 of the stress granules (SGs) with SuperNova-Red and inactivated the SGs using CALI technology to analyze the response mechanism and role of SGs in response to external pressure in cells.

## 6. Future Perspective of CALI

As previously mentioned, a crucial aspect of CALI technology is to find a suitable chromophore that can selectively induce damage to target proteins while preventing damage to neighboring proteins. The type of chromophore, its capacity for generating ROS, stability, and range of ROS production, as well as the sensitivity of target proteins towards these reactive oxygen species collectively influence the effectiveness of CALI [67]. For instance, if the distance between the chromophore and the functional domain of the target protein exceeds the range of oxygen radicals or if the target protein exhibits poor sensitivity towards oxygen free radicals, it is difficult to achieve the purpose of targeted inactivation of proteins. Moreover, if the range of oxygen radicals generated by the chromophore is excessively broad, it can potentially inflict damage not only on the target protein but also on neighboring proteins and even compromise cellular integrity. Another crucial aspect of CALI technology lies in how to achieve precise labeling of target proteins through chromophores. The labeling of target proteins with chromophores is primarily achieved through either antigen–antibody reactions or genetic engineering to create fusion protein labeling. However, the introduction of chromophores may impact the structure and function of the target protein, thereby potentially affecting its biological activity. Hence, not all proteins can be effectively labeled using current methods. Applications of CALI are frequently constrained by the options for chromophores and labeling techniques. With continuous advancements in both chromophore and protein labeling techniques, CALI is progressively gaining recognition as a prominent research methodology within the biomedical field.

The future trends of CALI technology development will likely be similar to immunofluorescence staining technology that allows simultaneous manipulations of proteins such as labeling, imaging, and inactivation. Some recent studies have demonstrated the photoluminescence properties and ROS-generating capabilities of ultrasmall metal nanoclusters (core < 2 nm), such as gold and silver nanoclusters [68], and methods using metal nanoclusters include using them alone or in conjunction with other photosensitizers to achieve ROS generation and imaging purposes. Feng et al. [69] realized photosensitizing and photo crosslinking functions of the synthesized fluorescent protein chromophore through the expansion of conjugation and introduction of heavy atoms. This novel fluorescent protein chromophore holds great potential for application in CALI technology. The aforementioned research posits that we can achieve new developments in CALI technology by synthesizing new photosensitizers through the expansion of research on ultra-small metal nanoclusters or conjugations and the introduction of structural modulation methods of heavy atoms.

In addition to the development of photosensitizers, the newest development direction in protein chemistry is known as selective site protein modification chemistry [70,71]. This field primarily focuses on modifying a specific chemical group to a known site of the protein through bioorthogonal chemistry. By employing this approach, direct protein modification can be achieved without compromising its functionality, thus ensuring precise and targeted modifications. Among them, because of screening from the random single-stranded nucleotide library by SELEX (Systematic Evolution of Ligands by Exponential Enrichment) technology [72], aptamers exhibit remarkable specificity and affinity with the target substance and can guide site-selective protein–DNA coupling [73]. For instance, Cui et al. [74] have reported an aptamer-templated synthesis (ATS) technique for precise site-selective protein/DNA recognition and conjugation at specific sites. As a demonstration model, they selected human α-thrombin and its specific aptamer HD22. They designed a segment of reacting oligonucleotide (RO) that exhibits partial complementarity to HD22 and a modified n-hydroxysuccinimide (NHS) ester at one end of the sequence. This modification enables efficient and rapid reaction with the primary amino group in a mild, weak alkaline aqueous solution. Under the affinity provided by HD22, RO can approach thrombin, leading to stable binding between thrombin and RO facilitated by a cross-linker agent. Finally, through the addition of cDNA that is fully complementary to HD22 for aptamer removal purposes, a protein–RO conjugate can be successfully obtained. In addition, there are many selective modifications based on peptides and protein sites that can also be used as hot spots for future CALI technology research and development [75,76,77]. It is believed that in the near future, with the continuous development of protein site-selective modification and aptamers, CALI can be achieved in any protein.

The current CALI technology is not as advanced as immunofluorescence technology, and consequently, it still faces numerous challenges that need to be addressed. However, CALI technology possesses a remarkable advantage in its ability to precisely manipulate specific inactivated proteins using optical precision. With ongoing technological advancements, it holds the potential for achieving high-throughput optical manipulation of multiple proteins in the future. This would enable a comprehensive understanding of the biological role of target proteins and genes, thereby playing an indispensable role across various domains within life sciences.

## Figures and Tables

**Table 1:** Category and characteristics of photosensitizers.

Category	Photosensitizer	Targeting	Characteristics
Chemical photosensitizers (CPSs)	Malachite green	Antibody	Laser and microinjection are needed.
	Fluorescein	Antibody	Ordinary light and microscopes are sufficient, ROS production is not high enough.
	Eosin	HaloTag	Singlet oxygen is 11-fold higher than fluorescein, and it produces a large amount of detectable fluorescence.
Genetically encoded photosensitizers (GEPSs)	Green fluorescent protein (GFP)	Fusion	ROS production is not high enough.
	KillerRed	Fusion	Severalfold more active than GFP, prone to homodimer molecules, may cause damage to mitochondria, cell membranes, and DNA and interfere with cell mitosis, eventually causing cell cycle arrest or cell death.
	SuperNova	Fusion	Same structural features and photosensitizing activity as KillerRed, but has higher folding and maturation efficiency in vitro, manifesting as monomeric proteins under physiological conditions.
	KillerOrange	Fusion	Another orange dimer variant obtained through random mutagenesis in KillerRed; prone to homodimer molecules.
	SuperNova-Green	Fusion	First green photosensitizer protein derived from KillerRed, a green monomer variant.
	SuperNova 2	Fusion	Produces enhanced brightness, chromophore maturity, and phototoxicity in bacterial and mammalian cell cultures.
	KillerRed 2	Fusion	Produces enhanced brightness, chromophore maturity, and phototoxicity in bacterial and mammalian cell cultures.
	MiniSOG	Fusion	Smallest green-emitting monomeric protein, a higher singlet oxygen production rate than the SuperNova, and it is a monomeric fluorescent protein that does not cause abnormal cell division.
